# Rational design of alternative treatment options for radioresistant rectal cancer using patient-derived organoids

**DOI:** 10.1038/s41416-025-02989-4

**Published:** 2025-04-10

**Authors:** D. Andel, A. J. Nouwens, S. Klaassen, J. Laoukili, B. Viergever, A. Verheem, M. P. W. Intven, M. Zandvliet, J. Hagendoorn, I. H. M. Borel Rinkes, O. Kranenburg

**Affiliations:** 1 Department of Surgical Oncology, University Medical Center Utrecht, Cancer Center, Utrecht, The Netherlands; 2 Laboratory for Translational Oncology, University Medical Center Utrecht, Cancer Center, Utrecht, The Netherlands; 3 Department of Radiation Oncology, University Medical Center Utrecht, Cancer Center, Utrecht, The Netherlands; 4 Department of Clinical Sciences, Faculty of Veterinary Medicine, Utrecht University, Utrecht, The Netherlands; 5 Utrecht Platform for Organoid Technology, Utrecht University, Utrecht, The Netherlands

**Keywords:** Drug development, Radiotherapy

## Abstract

**Background:**

Resistance to radiation therapy is a common challenge in the field of oncology. Cancer cells with an increased ability to effectively repair DNA or cells with higher levels of antioxidants are more resistant to radiation. As cancer cells rely on these traits for survival, they may offer vulnerabilities that could be exploited.

**Methods:**

In the current study, rectal cancer organoids that showed different responses to radiation treatment were identified. RNA sequencing was used to compare radioresistant and radiosensitive organoids. In vitro combination drug screens were performed. The selection of drugs was guided by the RNA sequencing results.

**Results:**

Radioresistant organoids exhibited superior transcriptional adaptability and activated more DNA repair pathways when irradiated. Additionally, radioresistant organoids displayed enhanced antioxidant metabolism, including pathways related to the detoxification of reactive oxygen species and the synthesis of glutathione. Combinatorial drug screens identified the combination of RRx-001 (an inducer of oxidative stress) with GCLC inhibitor BSO as a highly effective and synergistic drug combination in killing radioresistant organoids. CRISPR-CAS-mediated knockout of *GCLC* sensitised organoids to RRx-001.

**Conclusion:**

Combining RRx-001 with the inhibition of GCLC may be a promising alternative treatment strategy in radioresistant rectal cancer.

## Introduction

Radiation therapy plays a critical role in the treatment of cancers, but resistance remains a formidable challenge [1, 2]. Radiation induces cell death by ionizing DNA or indirectly by generating reactive oxygen species that damage DNA and other crucial cellular components [3]. The mechanisms by which cancer cells develop resistance to radiation depend on both cell non-autonomous factors and cell-autonomous factors. Critical cell non-autonomous factors include hypoxia, the presence of fibroblasts, and anti-cancer immune responses [4]. Meanwhile, cell-autonomous factors encompass proficient DNA repair processes and the rewiring of the cell’s metabolism to reduce reactive oxygen species levels and supply the necessary materials for effective repair of damaged cellular structures [5]. Resistance can be acquired by cancer cells through DNA mutations that modify gene function or copy number, as well as by the regulation of gene expression [6]. Because these traits provide a survival advantage to cancer cells when exposed to radiation, they may represent vulnerabilities that can be therapeutically exploited.

Cancer-derived organoid cultures are an interesting model for studying radioresistance, as they recapitulate the characteristics of the tumour they are derived from and can be passaged stably [7]. Recently, it was shown that in vitro sensitivity to irradiation correlates well with *clinical* response to radiation therapy in both rectal adenocarcinoma-derived organoids [8, 9], as well as organoids derived from head and neck squamous cell carcinoma [10, 11]. We previously demonstrated that primary rectal cancer-derived organoids display heterogeneous responses to radiation in vitro, following exposure to clinically relevant radiation doses [12]. It was also shown that the in vitro response to irradiation was associated with progression-free survival of the corresponding patient: patients with radioresistant organoids experienced progression after surgery earlier than patients with radiosensitive organoids.

In this present study, two radioresistant organoids and two radiosensitive organoids were selected from this cohort. Compared to radiosensitive organoids, radioresistant organoids exhibited upregulated antioxidant metabolism, including genes stimulating glutathione synthesis. Through drug screens that combined RRx-001, a novel inducer of oxidative stress [13–16], with various inhibitors of antioxidant metabolism, we identified GCLC inhibition as highly synergistic with RRx-001 in radioresistant organoids. This was validated genetically in GCLC knockout organoids. Thus, we have identified a synergistic drug combination therapy that could be employed to target radioresistant cancer cells as an alternative to radiotherapy.

## Methods

### Tumour organoid culturing

Patient-derived organoids were obtained following the HUB biobank protocol HUB-cancer TcBio#12-09, which was approved by the medical ethics committee at UMC Utrecht. All patients provided informed consent. Supplementary Table S1 details the clinical and mutational status of all organoids used. Patient-derived cancer organoids were cultured using previously established methods [7, 17]. The organoids were cultivated in a colorectal cancer culture medium of advanced DMEM/F12 (Invitrogen) supplemented with the additives outlined in Supplementary Table S2. Tumour organoids were passaged by dissociation with TrypLE (Gibco) for 5 min and embedded in a mixture of Basement Membrane Extract (BME; Amsbio) and culture medium in a 3:1 proportion. Subsequently, the tumour organoids were replated in 10 μl drops on a pre-warmed 6-well plate. To prevent anoikis, a concentration of 10 μM ROCK inhibitor (Y-27632, Tocris) was introduced to the culture medium for two days.

### Analysis of cell death by flow cytometry

Three days after passaging (three-day-old), organoids were exposed to radiation doses of either 0 Gy or 5 Gy delivered using a linear accelerator, specifically the Elekta Precise Linear Accelerator 11F49 or the Elekta Synergy Agility Linear Accelerator. To account for photon scattering effects, culture plates were positioned on a 2-cm polystyrene board. Five days later, the organoids were dissociated into single cells and suspended in 50 μl of cold phosphate-buffered saline. Cells were fixed by adding ice-cold 70% ethanol drop by drop and then incubated for 15 minutes at 4 °C. DNA was stained with 200 μl of propidium iodide, and RNase A (100 μg/ml) was added to degrade RNA molecules. The suspension was then incubated at 37 °C for 30 min.

Data were acquired using the FACSCelesta™ Cell Analyzer (BD Biosciences) and analysed with FACSDiva v.8.0.1.1 (BD Biosciences). Cell debris and doublets were excluded using SSC-A/FSC-A and FSC-A/FSC-H gates, respectively. Cell cycle phases were determined based on the 2N and 4N peaks by gating the histograms. The fraction of cells in the sub-G1 population, which indicates cell death, was quantified.

### Clonogenic survival assay

Three days after passaging, organoids were dissociated into single cells and suspended in a 150 µL mixture of BME and culture medium in a 2:1 ratio. A 150 µL droplet of this mixture, containing 1000 cells, was evenly spread in a well of a 6-well plate. Following a 30-minute incubation at 37 °C, the cells were irradiated at the indicated doses. Medium was refreshed weekly. Fourteen days post-irradiation, the wells were imaged at 4× magnification. A custom Fiji (ImageJ) macro script was employed to automate colony detection and counting. Shortly, a rolling ball background subtraction (radius 115 pixels) and Gaussian blur (sigma 2 pixels) were used, and after background subtraction and thresholding, colonies were detected based on the area (minimum size 5000 µm², ±50 cells) and circularity (≥0.5).

### Gene expression methodology, profiling, and analyses

Three-day-old organoids were exposed to either 0 Gy or 10 Gy of radiation to ensure a robust transcriptional response. An Elekta Synergy Agility Linear Accelerator was used. RNA was extracted from cells at day 0 (1 h before irradiation) and day 1 (24 h after irradiation). This allowed the comparison of radioresistant versus radiosensitive organoids at baseline (day 0, comparison A in Supplementary Fig. S2a) and at 24 h following radiation (comparisons B in Supplementary Fig. S2a), as well as the comparison of unirradiated versus irradiated organoids (comparisons C and D in Supplementary Fig. S2a). Comparisons A and B allow differential gene expression analyses between radioresistant and radiosensitive organoids at baseline (A), and 24 hours following irradiation (B). Comparison C and D measure how radioresistant (C) and radiosensitive (D) organoids respond to irradiation. For each organoid and each condition, three replicates were used.

RNA was extracted from cells using the RNeasy Plus Mini Kit (Qiagen) according to the manufacturer’s protocol and at the indicated time points. Generation of sequencing libraries was performed using the Truseq RNA stranded poly A Library Preparation Kit (Illumina, San Diego, CA, USA). Sequencing was performed on an Illumina NextSeq2000 (1 ×50 bp). Sequencing reads were mapped to human reference GRCh37 using STAR (v2.7.3a). Read counts were generated using featureCounts. Lowly expressed genes were filtered and the remaining reads were normalised using the trimmed mean of M-values [18] with the *edgeR* package (v. 3.15) [19]. Heteroscedasticity was corrected using *Voom* from the *limma* package (v. 3.56.2) [20]. Differential expression was analysed using *limma*. Gene set variation analysis was performed using the *GSVA* package (v. 1.48.3) [21]. Enrichment scores were generated for gene sets belonging to the KEGG [22], Reactome [23], Biocarta, and Hallmark [24] databases, all downloaded from the MsigDB database [24]. Gene set network plots were constructed using the *igraph* package with wrapper function from the *ggraph* package (v. 2.1.0.9), using the Distributed Recursive Graph Layout [25]. Benjamini–Hochberg (BH) was used to control the false discovery rate at 1%. Significantly differentially expressed genes and gene sets were used for the gene set-gene set plots. Transcriptional response rates of gene sets were quantified by computing $-{\log }_{10}\left({P}_{{BH\; adj}}\right)x{\log }_{2}({fold\; change})$ [26].

### Western blotting

Organoids were collected using dispase, rinsed with PBS, and then disrupted in Laemmli lysis buffer, which consisted of 2.5% SDS, 20% glycerol, and 120 mM TRIS at pH 6.8. Approximately 10–20 μg of protein was electrophoresed on SDS-PAA gels, followed by transfer onto nitrocellulose membranes (Trans-Blot Turbo, Bio-Rad, Hercules, CA, USA), and subsequently incubated with specific antibodies (refer to Supplementary Table S3 for specifics).

### Quantification of glutathione

To quantify the differences in total glutathione between radioresistant and radiosensitive organoids, 7000 single cells per technical replicate were plated in a 96-well plate, with or without 50 μM BSO. The following day, the total GSH was measured using the GSH/GSSG Glo Assay kit (Promega, V6611), following the manufacturer’s instructions. Luminescence was measured using a SpectraMax® M5e Series Multi-Mode Microplate Reader (Molecular Devices, MVE05640).

### CRISPR CAS9 *GCLC* knockout organoids

Ecas9 expressing Tor10 control and its CRISPR-Ecas9-engineered *GCLC* knockout (KO) variant (GCLC 24.1) were previously generated by our group [27]. The following target sequences were used:

gRNA-hGCLC-Oligo2-Forward: 5′CACC GTGTGCCGGTCCTTGACGGCG

gRNA-hGCLC-Oligo2-Reverse: 5′ AAAC CGCCGTCAAGGACCGGCACAC

Viability between Ecas9 control and GCLC knockout organoids was compared by plating out 3000 single cells in a 4 μL droplet containing 3 μL of BME and 1 μL of organoid in a 96-well plate. The organoids were grown overnight, and ATP levels were measured using CellTiter-Glo® 3D (Promega, G9681). For specific details, refer to the section ‘In vitro drug and radiation treatments’.

### In vitro drug and radiation treatments

On the day of the drug screen, organoids were harvested, dissociated with TrypLE (Gibco), and plated as 4 μL droplets, each containing 3 μL of BME and 1 μL of organoid medium, in a 96-well culture plate. Each droplet consisted of 3000 single cells. Next, 100 μL of reduced growth medium (DMEM/F-12, Sigma-Aldrich, containing no glutathione) with or without N-acetyl cysteine (NAC) was added to each well. Drugs, dissolved in either DMSO or Tween-20, were dispensed 1 h after adding the medium using the Tecan HP D300e Digital Dispenser (HP, USX1335002). All wells were normalised for solvent, ensuring that DMSO or Tween-20 percentages did not exceed 1% or 0.3%, respectively. After 72 h, the reduced growth medium and drugs were refreshed. Seven days after dissociation, organoid viability was measured using CellTiter-Glo® 3D (Promega, G9681), and luminescence was recorded using a SpectraMax® M5e Series Multi-Mode Microplate Reader (Molecular Devices, MVE05640). Viability assays were performed in triplicate or quadruplicate and repeated on multiple occasions. For a list of all drugs and their concentrations, refer to Supplementary Table S4.

In the in vitro radiation experiments, RRx-001 was dispensed 72 h before radiation, following the same procedure described earlier. On the day of irradiation, the culture medium and drugs were refreshed. Subsequently, organoids were irradiated with doses of 0, 2, or 4 Gy using either the Elekta Precise Linear Accelerator 11F49 or the Elekta Synergy Agility Linear Accelerator. A separate plate was used for each dose. To account for photon scattering, the plates were positioned on top of a 2-cm polystyrene board. Seven days after irradiation, organoid viability was measured.

### Determining synergy

Synergy was evaluated by comparing the observed combination response to the expected response. The expected response was determined using the Bliss independence metric [28]. According to Bliss’ definition, the expected cell viability upon the simultaneous administration of drugs A and B, denoted as Y_AB(E)_, is calculated as:

$${Y}_{A}{{{\rm{\cdot }}}}{Y}_{B}={Y}_{{AB}(E)}$$

Synergy is observed when the observed viability (*Y*_*AB(O)*_) is lower than Y_AB(E)._ To quantify synergy, the expected viability was divided by the observed viability. In this way, a synergy score of two indicates that the drug combination killed twice as many cells as expected. We categorised a synergy score between two and five as ‘synergistic,’ while a score above five was labeled ‘strongly synergistic.’ A drug combination within a given organoid was classified as synergistic only when at least two out of three repeated experiments showed a synergy score greater than two.

### Statistical analyses

Chronos essentiality scores from the Cancer Cell Line Encyclopedia (CCLE) were downloaded from https://depmap.org [29, 30]. We used the 22Q3 release. R version 4.2.1 was used for statistical analysis. Continuous variables were compared using the Student’s *t* test.

## Results

### Identifying radioresistant and radiosensitive rectal cancer patient-derived organoids

**Fig. 1:**
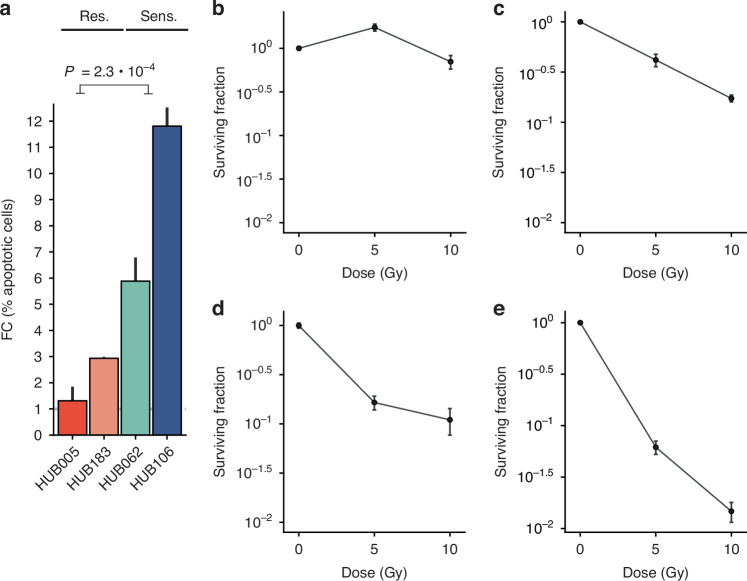
Flow cytometry-based measurement of (apoptotic) cells with sub-G1 DNA content at 0 and 5 Gy. **a** Fold change of the percentage of sub-G1 cells from 0 to 5 Gy. Barplots represent the mean ± sd of two independent experiments. Clonogenic assay survival curves for HUB005 (**b**), HUB183 (**c**), HUB062 (**d**), and HUB0106 (**e**). Dots are mean ± sem. One independent experiment was performed.

Previously, we screened rectal cancer-derived organoids for differential in vitro radiosensitivity using a Cell Titer Glo-based assay [12]. We selected from this study two of the most radioresistant organoids (HUB005 and HUB183) and two of the most radiosensitive organoids (HUB106 and HUB062) [12]. Tumour characteristics, clinical info, and mutation status are detailed in Supplementary Table S1. We performed two validation experiments to confirm the differential radiosensitivity of these organoids. First, the organoids were exposed to 5 Gy of radiation and apoptotic cells (with sub-G1 DNA content) were quantified by flow cytometry. This dose was chosen because it is well within the range that has been shown to correlate with clinical radiation responses [8, 9]. Radioresistant organoids (HUB005 and HUB183) showed a marginal increase in apoptotic cells (fold change 1.3 ± 0.5 and 2.9 ± 0.05, respectively), while radiosensitive organoids (HUB062 and HUB106) exhibited a significantly greater increase (fold change 5.9 ± 0.9 and 11.8 ± 0.7, respectively). (Fig. 1a, *P*-value = 2.3 ×10^−2^, Student’s *t* test, Supplementary Fig. S1a–c). Secondly, clonogenic survival assays showed similar differential sensitivities, with HUB005 and HUB183 retaining more clonogenic capacity than HUB062 and HUB106 (Fig. 1b–e, Supplementary Fig. S1d).

### Radioresistant organoids display increased transcriptional adaptability and DNA damage response following irradiation

To test whether radioresistant and radiosensitive organoids responded differently to irradiation, three-day-old organoids were exposed to 0 or 10 Gy of radiation. RNA was isolated from all organoids and bulk RNA sequencing was performed. (Methods and Supplementary Fig. S2a). Gene set variation analysis was performed for gene sets belonging to the KEGG [22], Reactome [23], Biocarta, and Hallmark [24] databases. In this analysis, 469 gene sets were significantly up-or downregulated in radioresistant organoids in response to irradiation, whereas 145 gene sets were significantly up- or downregulated in radiosensitive organoids (Supplementary Fig. S2b, Supplementary Tables S5 and S6). Subsequently, all the differentially expressed gene sets were plotted on a ‘gene set-gene set’ graph using a force-directed graph algorithm [25]. This algorithm is employed to visualise gene sets as nodes and positions gene sets that share many genes closer together, thus facilitating the identification of gene sets with similar functions.

This analysis revealed that radioresistant organoids significantly upregulated 43 DNA repair pathways (out of 469 significantly differentially expressed gene sets in total) (Supplementary Fig. S2c) [31]. The upregulated DNA repair pathways included those associated with double-strand break repair, such as non-homologous end joining and homologous recombination (Supplementary Fig. S2d). Radioresistant organoids also upregulated gene sets involved in cell cycle regulation (87 out of 469) such as M to G1 and G1 to S transition (upregulated), as well as mitosis-associated gene sets such as the resolution of sister chromatid cohesion and regulation of mitotic cell cycle (both upregulated). Furthermore, radioresistant organoids upregulated gene sets involved in the regulation of transcription (19 out of 469, e.g., gene sets involved in RNA polymerase I transcription termination and mRNA capping).

In contrast, radiosensitive organoids exhibited fewer upregulated DNA repair pathways in response to irradiation (16 out of 145), as well as cell cycle pathways (19 out of 145). Moreover, they upregulated pathways associated with programmed cell death (11 out of 145, Supplementary Fig. S2e,f, Supplementary Table S6). Finally, quantification of the transcriptional responses (Methods) revealed that the DNA repair pathways were significantly more strongly upregulated in radioresistant than in radiosensitive organoids (Supplementary Fig. S2g, *P* = 5.4 ×10^−6^).

These results link the differential sensitivities to irradiation of the organoids with the enrichment of gene sets known to be associated with radiation sensitivity [5] and indicate that radioresistant organoids have superior transcriptional adaptability in response to irradiation.

### Radioresistant organoids have upregulated metabolism associated with ROS detoxification

**Fig. 2:**
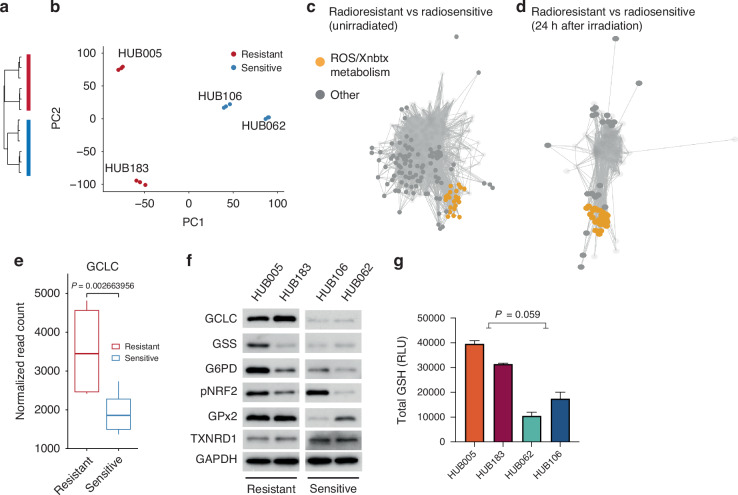
Radioresistant organoids have higher glutathione metabolism than radiosensitive organoids. Principal component analysis (**a**) and unsupervised clustering (**b**) of radioresistant and radiosensitive organoids based on bulk RNA expression data. **c** Gene set-gene set graph of differentially expressed gene sets (false discovery rate controlled at 1%, using Benjamini–Hochberg) between unirradiated radioresistant and unirradiated radiosensitive organoids. Each dot indicates an MSigDB gene set. Gene sets that share many genes will cluster together and thus have similar functions. Gene sets are together by vertices, whose thickness is proportional to the number of genes shared between gene sets. Opaque dots indicate upregulated gene sets in radioresistant organoids, while translucent dots indicate downregulated gene sets. **d** Gene set-gene set graph of differentially expressed gene sets between irradiated radioresistant and irradiated radiosensitive organoids. **e** Gene expression of *GCLC* in unirradiated radioresistant organoids and unirradiated radiosensitive organoids. **f** Western blot of indicated proteins in unirradiated radioresistant and radiosensitive organoids. **g** Luminescence measurements of total glutathione levels of unirradiated radioresistant vs. radiosensitive organoids. Three independent experiments were performed.

While the previous analysis focused on how organoids respond to irradiation, distinctions between radioresistant and radiosensitive organoids may also exist inherently before radiation. These differences may persist or change upon irradiation. These inherent differences may be more easily translated into actionable treatments, as they can be identified before administering radiation therapy. Therefore, a comparison of expression profiles between unirradiated radioresistant and unirradiated radiosensitive organoids was made. Hierarchical clustering (Fig. 2a) and principal component analysis (Fig. 2b) of genome-wide expression data separated radioresistant and radiosensitive organoids into distinct clades and groups, respectively. The analysis of differential gene expression between these two groups identified 4,093 differentially expressed genes (Supplementary Table S7). Notably, the top upregulated genes in radioresistant organoids included several genes associated with detoxification, such as *GSTT1* (involved in the conjugation of reactive oxygen species with glutathione), *CYP1A1* (participating in detoxification of xenobiotic compounds), *UGT1A6* (associated with glucuronidation), and *ALDH3A1* (involved in acetaldehyde detoxification) (Supplementary Table S7). Gene set variation analysis and gene set-gene-set graph analysis indicated that radioresistant organoids exhibited increased expression of gene sets related to the detoxification of reactive oxygen species (ROS) and xenobiotics, including pathways such as glutathione metabolism, nicotinate, nicotinamide, and ascorbate metabolism (Fig. 2c, Supplementary Table S8). Furthermore, these distinctions between radioresistant and radiosensitive organoids were even more pronounced after irradiation (Fig. 2d, Supplementary Table S9), with ‘Glucuronidation’ and ‘Glutathione synthesis and recycling’ being the most differentially expressed gene sets, primarily upregulated in radioresistant organoids.

We next focused on glutathione metabolism, as glutathione is one of the most important antioxidants in cells. *GCLC*, the rate-limiting enzyme in glutathione (GSH) synthesis, was found to be upregulated in unirradiated radioresistant organoids compared to unirradiated radiosensitive organoids (Fig. 2e, Benjamini–Hochberg (BH)-adjusted *P*-value = 2.7 ×10^−3^). Moreover, western blot analyses revealed strong protein expression of GCLC in radioresistant organoids HUB005 and HUB183, but not in radiosensitive organoids HUB106 and HUB062 (Fig. 2f). Finally, radioresistant organoids had higher levels of GSH, although this difference was not statistically different (Fig. 2g, Student’s *t* test, *P* = 0.059).

### Identification of a synergistic combination therapy for radioresistant organoids

**Fig. 3:**
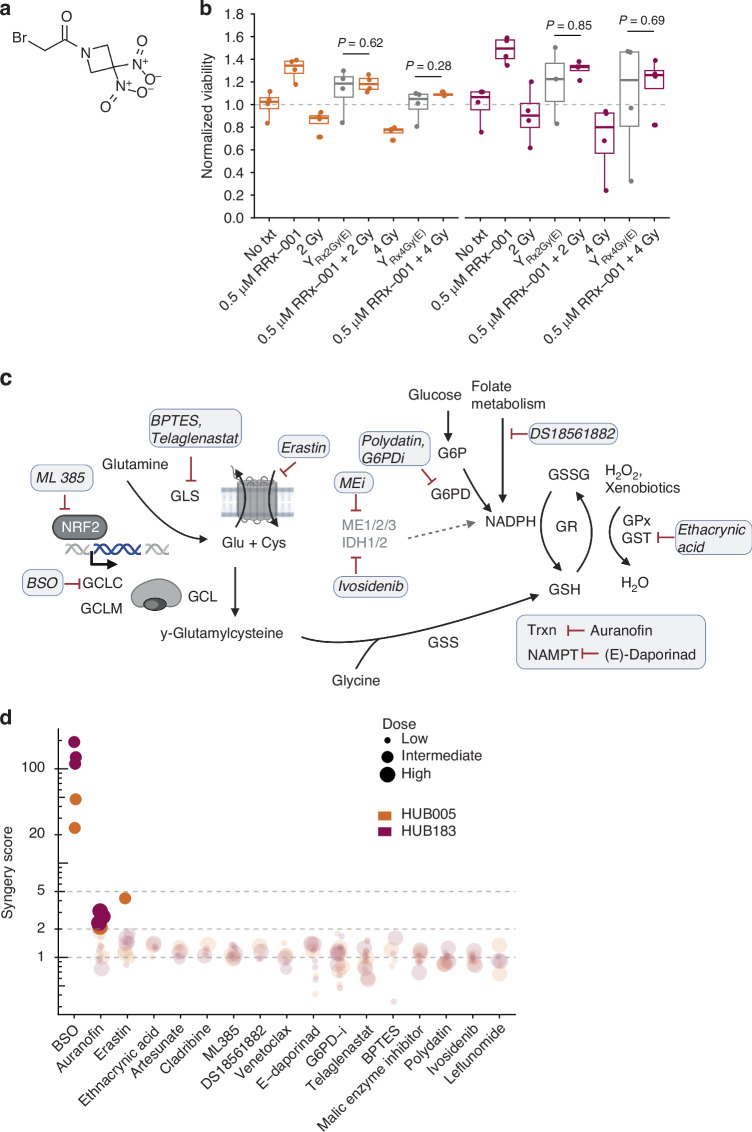
RRx-001 combination screen identifies RRx-001 and BSO as synergistic drug combination. **a** Chemical formula of RRx-001. **b** Viability of radioresistant organoids HUB005 and HUB183 at indicated RRx-001 concentrations and radiation doses. The conditions are normalised to DMSO-treated conditions (0 μM RRx-001; 0 Gy). Y_Rx2Gy(E)_ / Y_Rx4Gy(E)_: Bliss’ expected viability combining 0.5 μM and 2 or 4 Gy, respectively (Methods). **c** Schematic illustrating the mode of action of the screened drugs. **d** Dot plot showing synergy scores of the tested compounds with 20 μM BSO. Size reflects the concentrations of the compounds (Supplementary Table S4). Cys Cysteine, GCLC/GCLM Glutamate-cysteine ligase catalytic/modifier subunit, GSS Glutathione synthetase, GR Glutathione reductase, GSSG Glutathione disulfide, GSH Glutathione, GLS Glutaminase, GPx Glutathione peroxidase, G6P(D) Glucose-6-phosphate (dehydrogenase), IDH1/2 Isocitrate dehydrogenase 1/2, NADPH Nicotinamide adenine dinucleotide phosphate, NRF2 nuclear factor erythroid 2–related factor, NAMPT Nicotinamide phosphoribosyl transferase, Trxn Thioredoxin reductase, ME1/2/3 Malic enzyme 1/2/3. At least three independent experiments were performed for each drug combination and each organoid.

The above results suggest that radioresistant organoids may have a high capacity to resolve oxidative stress. An exciting new drug, RRx-001, a first-in-class dinitroazetidine (Fig. 3a), is a potent inducer of oxidative stress in cancer cells by forming covalent adducts with thiol sulfurs. As such, it depletes cellular stores of thioredoxin, cysteine, and glutathione [16, 32, 33]. RRx-001 has antitumour activity in several cancer types and is currently evaluated in multiple clinical trials [13–15]. As radioresistant organoids had upregulated detoxification metabolism, it was hypothesised that combining RRx-001 with irradiation could overwhelm the cells’ capacity to cope with oxidative stress. However, pretreatment of radioresistant organoids with RRx-001 failed to synergise with irradiation in killing radioresistant organoids (Fig. 3b).

Because radioresistant organoids had increased glutathione metabolism compared to radiosensitive organoids, we evaluated whether RRx-001 could synergistically induce cell death when combined with inhibitors of GSH or GSH-related metabolism such as buthionine sulfoximine (BSO), erastin and telaglenastat. Other inhibitors of redox metabolism and inhibitors of pathways involved in the production of NADPH, such as polydatin and malic enzyme inhibitor (MEi), were also included, as well as agents affecting purine and pyrimidine metabolism, and the apoptotic activator venetoclax. In total, 16 drug combinations were tested for their effect on both radioresistant organoids (HUB005 (*TP53, APC* mutant) and HUB183 (*TP53* mutant), Fig. 3c, Supplementary Table S1). For each combination in each organoid, a ‘Synergy Score’ was calculated to reflect the effectiveness of the combination compared to the expected cell death if the agents were to act independently (Methods). A synergy score of two, indicating that the combination killed twice as many cells as expected, was defined as synergistic, while a score of more than five was considered strongly synergistic. Most RRx-001-based drug combinations did not exhibit a synergistic effect (Fig. 3d). This observation aligns with recent extensive anti-cancer drug combination screens showing that synergy is rare [34–36]. However, the combination of RRx-001 with BSO, a selective inhibitor of Glutamate—cysteine ligase catalytic subunit (GCLC), displayed an extremely strong synergistic effect in both HUB005 and HUB183 (synergy score 113 [193–23.6], Fig. 3d).

**Fig. 4:**
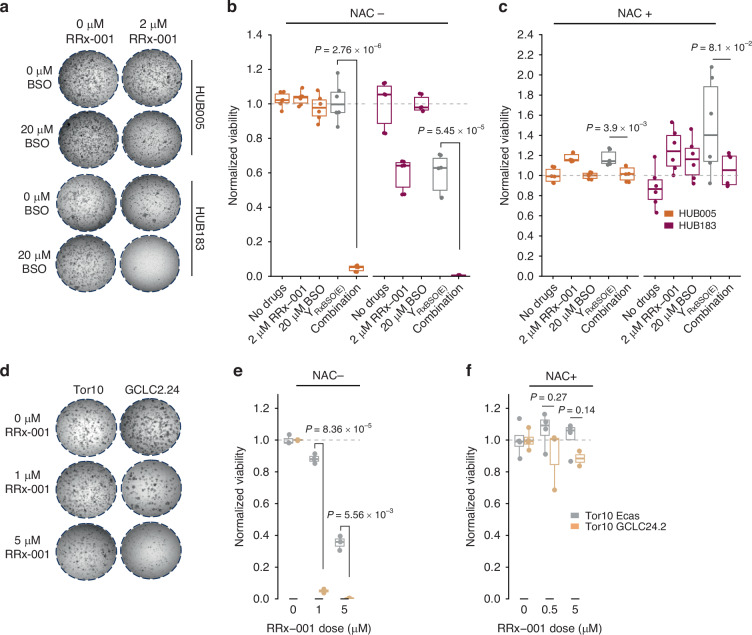
GCLC inhibition induces synergistic cell kill when combined with RRx-001. **a** Representative images of radioresistant organoids HUB005 and HUB183 after treatment with the indicated concentrations and drugs. Viability of radioresistant organoids at indicated RRx-001 concentrations, normalised to DMSO-treated conditions (0 μM RRx-001; 0 μM BSO) without (**b**) or with (**c**) the presence of N-acetyl cysteine (NAC). Y_RxBSO(E):_ Bliss’ expected viability for RRx-001 and BSO combined. **d** Representative images of Tor10 Ecas9 control and Tor10 *GCLC* knockout organoids. Organoids were treated with DMSO or with the indicated concentrations of RRx-001. Luminescence measurements of ATP levels (using CellTiter-Glo 3D) at indicated RRx-001 concentrations, normalised to DMSO-treated conditions (0 μM RRx-001) without (**e**) or with (**f**) the presence of N-acetyl cysteine (NAC). Three independent experiments were performed.

Next, it was tested whether BSO or RRx-001 affected the cell viability at the concentrations used in the screen. While BSO strongly decreased GSH levels in radioresistant organoids HUB005 and HUB183 (Supplementary Fig. S3a), it was ineffective in inhibiting cell viability alone, even at doses as high as 100 μM (Supplementary Fig. S3b). RRx-001 alone had no apparent effect on cell viability for HUB005, while it reduced viability by approximately 50% in HUB183 (Fig. 4a, b). However, combining 20 μM BSO with 2 μM RRx-001 induced strong synergistic cell death in both HUB005 and HUB183, causing a near-complete eradication of these radioresistant organoids (Fig. 4a, b). The addition of N-acetyl cysteine (NAC) to the medium, an antioxidant widely used to counteract oxidative stress [37], effectively attenuated the synergistic effect of BSO and RRx-001 (Fig. 4c). Next, a CRISPR-Ecas9-engineered *GCLC* knockout (KO) variant of the colorectal cancer-derived organoid Tor10 (GCLC 24.1, Figure S3c) was used and compared to control Tor10 organoids that only expressed Ecas9 [27]. As previously described, *GCLC* KO alone did not interfere with cell viability (Supplementary Fig. S3d, e) [27]. While Tor10 Ecas9 control was insensitive to 1 μM of RRx-001 and retained viability even at 5 μM, *GCLC* KO organoids were nearly eradicated by treatment with 1 μM and 5 μM RRx-001 (Fig. 4d, e). The addition of NAC to the medium attenuated the effect of RRx-001 in *GCLC* KO organoids (Fig. 4f).

### Combining buthionine sulfoximine with RRx-001 is synergistic in a set of colon cancer-derived organoids

**Fig. 5:**
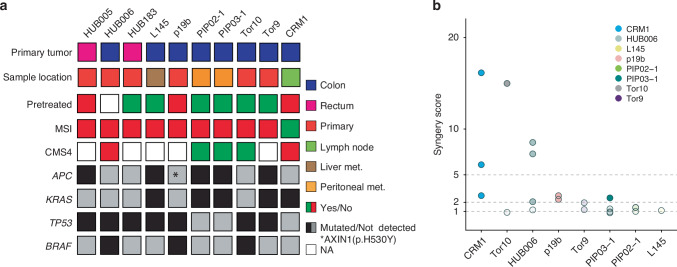
The BSO-RRx-001 is synergistic in other colorectal cancer-derived organoids. **a** Heatmap showing the characteristics of the organoids for which the BSO RRx-001 combination was tested. P19b has no APC mutation but is mutated in the wnt-related gene *AXIN1*. **b** Dot plot indicating synergy score for each tested organoid using 20 μM BSO and 2 μM RRx-001. A synergy score of x indicates the drug combination killed x times more cells than expected (Methods). Translucent dots indicate experiments in which the drug combination was not synergistic (synergy score <2). Each dot is an independent experiment.

To test whether the drug combination was synergistic in other aggressive colorectal cancer subtypes, we evaluated it on a panel of organoids derived from liver, peritoneal, and lymph node metastases; pretreated vs. chemoradiation-naïve tumours; microsatellite unstable tumours; cancers of consensus molecular subtype 4 (CMS4); and *KRAS-* and *BRAF*-mutated tumours (Fig. 5a). The combination therapy was found to be synergistic or strongly synergistic in three out of eight organoids (CRM1, HUB006, and p19b, Fig. 5b). Notably, two of these organoids carried the *BRAF* V600E mutation (HUB006 and p19b).

## Discussion

In the present study, a rational approach was taken to identify alternative treatment options for radioresistant rectal cancer. Radioresistant organoids had superior transcriptional adaptability in response to irradiation and displayed high expression of DNA repair and antioxidant metabolism pathways.

A recent study demonstrated that the genome-wide selection of transcription start sites has direct consequences for radiotherapy sensitivity [38]. Transcripts initiated from pyrimidine:cytosine (YC) dinucleotide motifs were enriched in radiotherapy-sensitive tumour samples and organoids, while transcripts initiated from pyrimidine:purine (YR) dinucleotide motifs were associated with resistance. They further showed that radiosensitization by PI3K/mTOR inhibition shifted the transcription start site repertoire from YR- to YC-initiated transcripts. These results link dynamic transcription start site utilization to radiotherapy sensitivity. As such, these mechanisms are likely to be highly relevant for the observed differences in transcriptional states and adaptability correlated with radiosensitivity that we have identified in the current study. Future work should therefore focus on linking these features to differences in transcription start site utilization by resistant versus sensitive organoids and to changes in transcription start site utilization after irradiation. Finally, mTOR inhibition in combination with RRx-001/GCLC-targeting may provide a powerful radio sensitization strategy.

Interestingly, the DNA damage response may increase antioxidant metabolism via increased production of the reducing agent NADPH, which results from upregulation of glucose-6-phosphate dehydrogenase (G6PD) through the ATM/ATR pathway [39–41]. We surmise that GSH synthesis is an inherent trait of radioresistant organoids and is not subject to the rapid metabolic rewiring needed to process DNA damage. In vitro combination drug screens identified RRx-001 and inhibition of GCLC as a highly effective and synergistic combination treatment in killing radioresistant rectal cancer organoids. Strikingly, this was not the case for the combination of RRx-001 with other inhibitors of reactive oxygen species (ROS) scavengers (e.g., Auranofin) or other compounds affecting glutathione synthesis, such as Telaglenastat or Erastin.

RRx-001 inhibits thiol-dependent redox metabolism by forming adducts to thiol sulfurs [16, 32, 33, 42]. It has a favourable toxicity profile and effectively induces tumour cell death as a standalone treatment or in combination with platinum-based therapies [13–15]. In line with our findings, the combination of RRx-001 and BSO, but not the single drugs, was also highly effective against leukaemia-derived cell lines [16].

Awaiting future research conducted in vivo, we envision that the combination of RRx-001 with GCLC-inhibition could be employed to target radioresistant cancers, for example in patients with unsatisfactory response to radiation therapy. This combination may have value in the context of radioembolization (RE), especially if it proves successful in metastatic colorectal adenocarcinoma and non-colorectal cancers. RE is a procedure for local‘ radiation treatment of hepatic tumours/metastases that involves the administration of radioactive microspheres into the hepatic arteries [43], the RRx-001/GCLC inhibition combination could be administered locally during the mandatory scout procedure, typically three to four days before the actual radiation treatment. Another area of potential clinical benefit involves microsatellite stable colorectal cancers with *BRAF*^V600E^ mutations. These tumours display aggressive behaviour and poorly respond to standard systemic therapies [44, 45]. The RRx-001/GCLC inhibition combination was highly effective in eradicating 2 out of 3 *BRAF*-mutant organoids in this study and may therefore represent an interesting alternative treatment option, even without radiation therapy [27].

We acknowledge several limitations in this study. Firstly, the RRx-001/GCLC-inhibition combination was tested on organoids in vitro, and future studies should assess its effectiveness in vivo. Moreover, although the combination therapy was designed rationally based on transcriptional differences between radioresistant and radiosensitive organoids, it was not evaluated whether these differences indeed were associated with either response to radiation or response to the combination therapy. Future studies should thoroughly investigate which tumour characteristics predict response to the combination therapy. Finally, while the combined inhibition of GCLC with RRx-001 clearly shows promise, intravenous BSO monotherapy has yielded only modest clinical tumour responses [46]. Furthermore, BSO has a relatively short half-life of just 2 h and fails to effectively reduce intratumoural GSH levels in cancer patients [47, 48]. Thus, the imperative is now to develop potent GCLC inhibitors, whether derived from BSO as a lead compound or via alternative strategies. The combination of effective GCLC inhibitors, in combination with RRx-001 could have a major impact on the treatment of various aggressive (colorectal) cancer subtypes, including radioresistant rectal cancer.

## Supplementary information

Legends for Supplementary Figures and Tables

Supplementary Tables.

Supplemental Figure 1. Gating strategies for cell death analyses and images from the clonogenic survival assays.

Supplemental Figure 2. Radioresistant organoids have increased transcriptional adaptability to irradiation

Supplemental Figure 3. Inhibition of GCLC alone is not effective in inducing cancer cell death.

## Data Availability

The raw sequencing data from this study are available in the NCBI Sequence Read Archive under the accession number PRJNA1198626. Processed data will be shared by the lead contact upon reasonable request.
