# Supplementary material for: Rational design of alternative treatment options for radioresistant rectal cancer using patient-derived organoids
Source: Br J Cancer. 2025 Apr 10;132(10):973–81. doi: 10.1038/s41416-025-02989-4 (PMC12081638; doi:10.1038/s41416-025-02989-4)
Supplement: Supplementary file 1 — Legends for Supplementary Figures and Tables [file 41416_2025_2989_MOESM1_ESM.docx]

**Legends for Supplementary Figures and Tables.**

**Figure S1. Gating strategies for cell death analyses and images from the clonogenic survival assays.**

**a**, Example of gating strategy for HUB005 at 0 and 5 Gy. **b**, Example of gating strategy for HUB106 at 0 and 5 Gy. **c**, Barplot indicating relative cell proportions in each cycle at the indicated doses. Sub-G1 cells represent cells in apoptosis. **d**, Representative images from the clonogenic survival assays conducted on organoids HUB005, HUB183, HUB062, and HUB106.

**Figure S2. Radioresistant organoids have increased transcriptional adaptability to irradiation**

**a,** Schematic illustrating the methodology of the RNA sequencing experiment (see also Methods). **b,** Differentially expressed gene sets before and after irradiation for radioresistant (red) vs. radiosensitive (blue) organoids. Each dot indicates an MSigDB gene set. Dots above the horizontal line indicate differentially expressed gene sets (BH-adjusted at a false discovery rate of 1%); 469 gene sets in radioresistant and 145 gene sets in radiosensitive organoids). **c,** Gene set-gene set graph of differentially expressed gene sets between irradiated and unirradiated radioresistant organoids. Opaque dots indicate upregulated gene sets (after irradiation), while translucent dots indicate downregulated gene sets. **d,** Bar plot showing all differentially upregulated gene sets with function in DNA repair in response to radiation in radioresistant organoids. **e,** Gene set-gene set graph of differentially expressed gene sets between irradiated and unirradiated radiosensitive organoids. **f,** Bar plot showing all differentially upregulated gene sets with function in DNA repair in response to radiation in radioresistant organoids **g,** Transcriptional response of DNA repair pathways that were upregulated after irradiation in radiosensitive (*n* = 16 pathways) versus radioresistant organoids (*n* = 43 pathways).

**Figure S3. Inhibition of GCLC alone is not effective in inducing cancer cell death.**

**a**, Total glutathione levels as measured by luminescence for HUB005 and HUB183 at 0 or 50 μM BSO. **b**, Viability of radioresistant organoids HUB005 and HUB183 at 20 and 100 μM BSO, normalised to control (DMSO). **c,** Western blot showing GCLC protein expression in Tor10 Ecas9 control and Tor10 *GCLC* knockout. **d**, Viability (as measured by ATP through CellTiter Glo 3D) of Tor10 GCLC, normalised to Tor10 Ecas9 control. Measurements were taken during the exponential phase of organoid growth. **e**, Density plot of Chronos essentiality scores for *GCLC*, *MYC,* and *KRAS* CRIPSR knockout across 1095 cancer cell lines. Lower scores indicate a gene is essential for the cell’s survival while a score of 0 indicates a gene is not essential. Cancer driver genes *MYC* and *KRAS* are highly essential, while *GCLC* is not.

Table Legends

**Table S1. Clinical data and mutation status of organoids.**

**Table S2. Supplements used for the colorectal cancer organoid medium.**

**Table S3. Antibody list.**

**Table S4. List of drugs used in drug screens.**

**Table S5. Differentially expressed gene sets comparing radioresistant organoids before and after irradiation.** Comparison C in Figure S2.

**Table S6. Differentially expressed gene sets comparing radiosensitive organoids before and after irradiation.** Comparison D in Figure S2.

**Table S7. Differentially expressed genes comparing unirradiated radioresistant versus unirradiated radiosensitive organoids.** Comparison A in Figure S2.

**Table S8. Differentially expressed gene sets comparing unirradiated radioresistant versus unirradiated radiosensitive organoids.** Comparison A in Figure S2.

**Table S9. Differentially expressed gene sets comparing irradiated radioresistant versus irradiated radiosensitive organoids.** Comparison B in Figure S2.
