# Supplementary figures and images for: Rational design of alternative treatment options for radioresistant rectal cancer using patient-derived organoids
Source: Br J Cancer. 2025 Apr 10;132(10):973–81. doi: 10.1038/s41416-025-02989-4 (PMC12081638; doi:10.1038/s41416-025-02989-4)

**a**

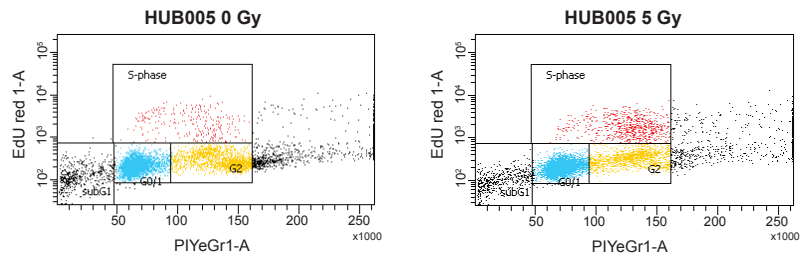

**b**

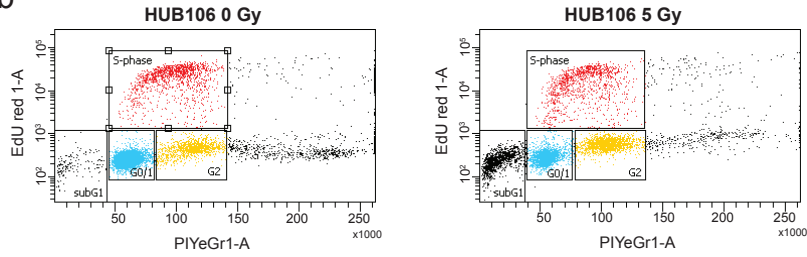

**d**

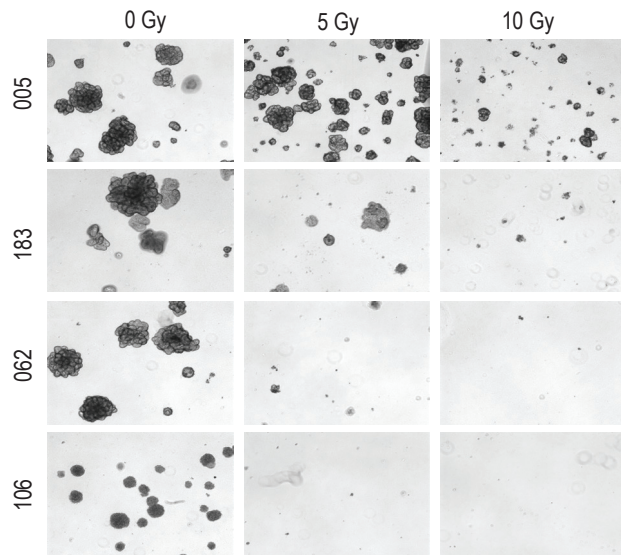

**c**

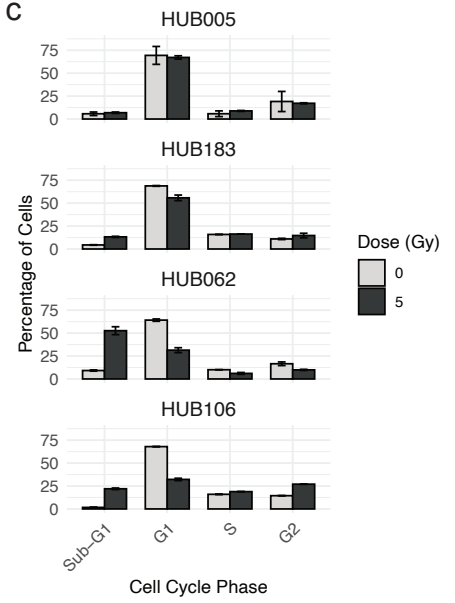

Supplement: Supplementary file 3 — Supplemental Figure 1. Gating strategies for cell death analyses and images from the clonogenic survival assays. [file 41416_2025_2989_MOESM3_ESM.pdf]

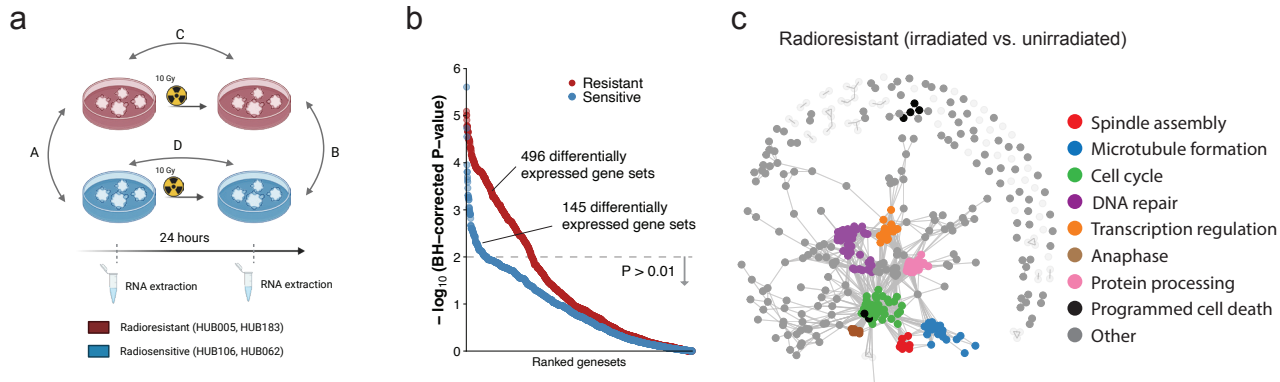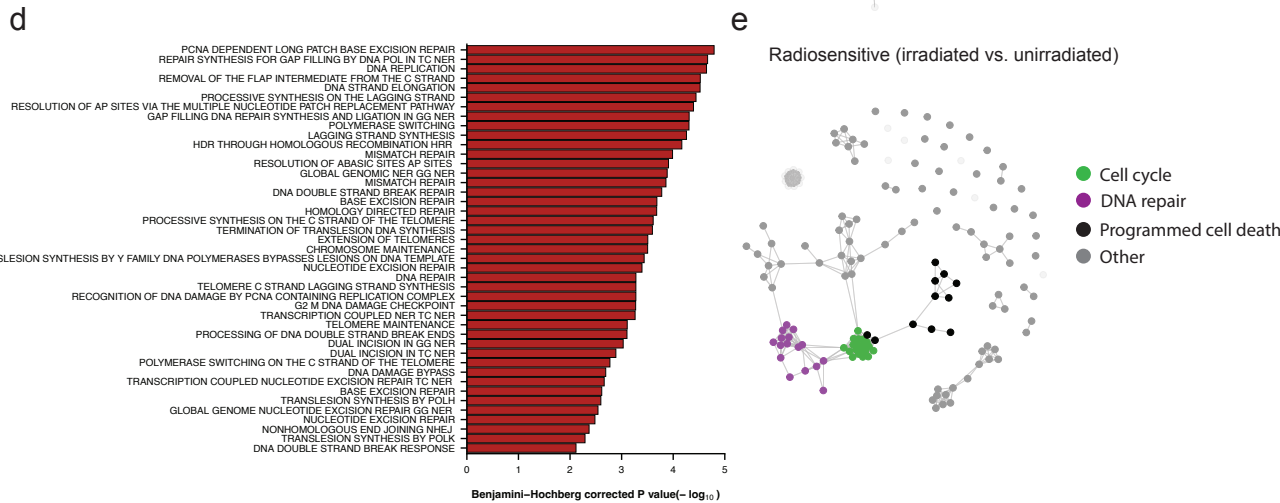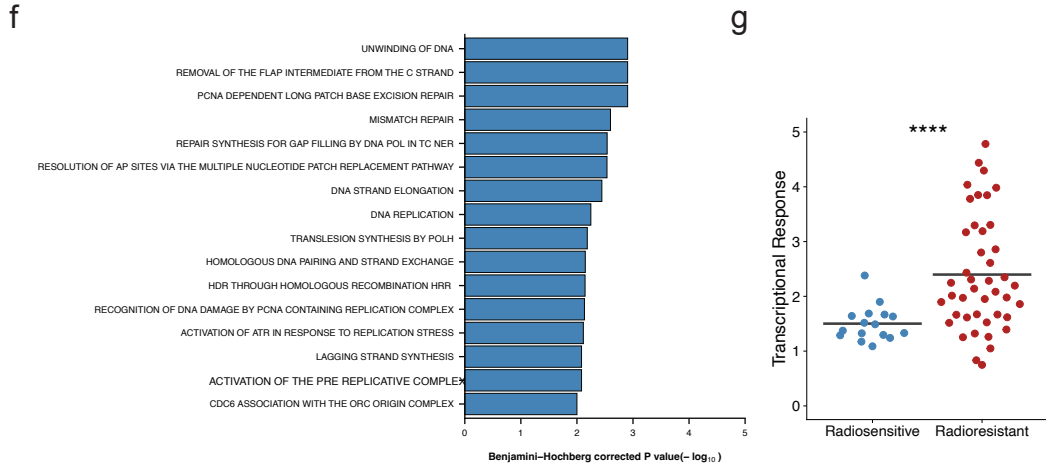

Supplement: Supplementary file 4 — Supplemental Figure 2. Radioresistant organoids have increased transcriptional adaptability to irradiation [file 41416_2025_2989_MOESM4_ESM.pdf]

a

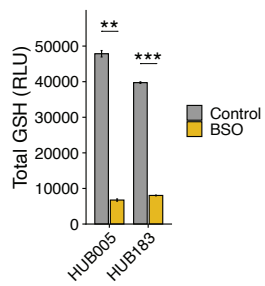

b

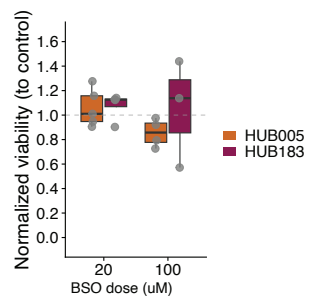

c

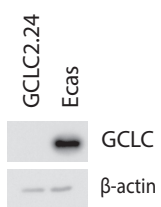

d

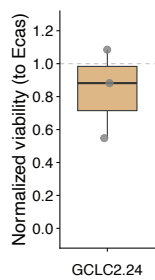

e

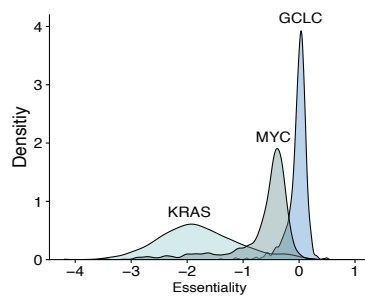

Supplement: Supplementary file 5 — Supplemental Figure 3. Inhibition of GCLC alone is not effective in inducing cancer cell death. [file 41416_2025_2989_MOESM5_ESM.pdf]
